# Current Murine Models and New Developments in H3K27M Diffuse Midline Gliomas

**DOI:** 10.3389/fonc.2019.00092

**Published:** 2019-02-27

**Authors:** John P. Welby, Tatiana Kaptzan, Anton Wohl, Timothy E. Peterson, Aditya Raghunathan, Desmond A. Brown, Shiv K. Gupta, Liang Zhang, David J. Daniels

**Affiliations:** ^1^Mayo Clinic School of Medicine, Mayo Clinic, Rochester, MN, United States; ^2^Department of Neurology, Mayo Clinic, Rochester, MN, United States; ^3^Department of Neurosurgery, Chaim Sheba Medical Center, Tel-HaShomer, Ramat-Gan, Israel; ^4^Department of Neurosurgery, Mayo Clinic, Rochester, MN, United States; ^5^Department of Laboratory Medicine and Pathology, Mayo Clinic, Rochester, MN, United States; ^6^Department of Radiation Oncology, Mayo Clinic, Rochester, MN, United States

**Keywords:** glioma, DIPG, diffuse intrinsic pontine glioma, H3K27M, xenograft

## Abstract

Diffuse Midline Gliomas with Histone 3-Lysine-27-Methionine (H3K27M) mutation constitute the majority of Diffuse Intrinsic Pontine Glioma (DIPG), which is the most aggressive form of pediatric glioma with a dire prognosis. DIPG are lethal tumors found in younger children with a median survival <1 year from diagnosis. Discovery of the characteristic H3K27M mutations offers opportunity and hope for development of targeted therapies for this deadly disease. The H3K27M mutation, likely through epigenetic alterations in specific H3 lysine trimethylation levels and subsequent gene expression, plays a significant role in pathogenesis of DIPG. Animal models accurately depicting molecular characteristics of H3K27M DIPG are important to elucidate underlying pathologic events and for preclinical drug evaluation. Here we review the past and present DIPG models and describe our efforts developing patient derived cell lines and xenografts from pretreated surgical specimens. Pre-treated surgical samples retain the characteristic genomic and phenotypic hallmarks of DIPG and establish orthotopic tumors in the mouse brainstem that recapitulate radiographic and morphological features of the original human DIPG tumor. These models that contain the H3K27M mutation constitute a valuable tool to further study this devastating disease and ultimately may uncover novel therapeutic vulnerabilities.

## Introduction

Brain tumors are the leading cause of cancer death in children. About 10% of pediatric brain tumors are primary brainstem tumors classified into four categories: diffuse, focal intrinsic, focal exophytic, and cervico-medullary glioma (1). Unfortunately, one of the most common form of brainstem glioma is diffuse intrinsic pontine glioma (DIPG), an aggressive tumor type found in young children. The median age at diagnosis for patients with DIPG is ~5–9 years and ~ 300 cases are reported each year in the United States (2, 3). With no effective therapy, DIPG remains lethal with <1 year median survival and <10% survive beyond 2 years (4, 5).

Numerous clinical trials have explored therapies for diffuse midline gliomas; none however, have extended the lifespan of these children beyond radiotherapy (2, 6). Recent advances demonstrate that H3K27M mutations including *H3F3A* and the less common *HIST1H3B* mutations contribute significantly to the pathogenesis of DIPG through alteration of H3K27 methylation status and subsequent gene expression (7–12). Given such findings correlating with their unique rarity and mortality, the World Health Organization now classifies these tumors as diffuse midline gliomas with the H3K27M and a grade IV tumor (13).

Unlike other tumor types, the rare occurrence and eloquent location within the brainstem make obtaining DIPG tissue difficult and have hampered previous research efforts due to a paucity of tissue. Now, as we start to unravel the genetic and epigenetic underpinnings of this disease, it has become extremely important to develop new model systems that reflect this unique biology. Here we describe various murine models for DIPG research and outline our experiences establishing new patient-derived DIPG animal models.

## Current Models

Previously, biopsy of brain stem gliomas was foregone for safety concerns, however, recent studies have demonstrated biopsies to be safe and useful to assess pathogenic mutations and for improving our understanding of tumor biology (14–17). Although the rate of success is moderately low (55% and 62%) for cell-derived orthotopic xenograft (CDOX) and patient biopsy-derived orthotopic xenograft (PDOX) model development, correspondingly (18), the surgically excised tissues (biopsy or autopsy) have been frequently used to develop DIPG models (19). While fresh tissue is preferred, the diffuse nature and pontine location often precluded safe biopsy, thus previous patient derived models have relied more on postmortem tissue (19–21). It is likely that models established from autopsies have prior exposure to treatment (including radiation and chemotherapy) that modifies the genetic and epigenetic features of DIPG tumors and, adding to the poor success rate, the quality of the autopsy tissue often exhibits significant degeneration (20–22). Considering that H3K27M and *TP53* mutations arise early in disease pathogenesis, secondary hits such as *ACVR1* may drive tumorigenesis while mutations in *PIK3CA* may be responsible for resistance to therapy and may arise later (23). Therefore, studies that investigate the terminal state of the disease and resistance mechanisms may benefit from autopsy derived cell models. In contrast, biopsy samples consist of early stages of tumor formation and are less likely to have treatment exposure and may better reflect events involved in tumor initiation (19, 24). Although biopsy tissue may reflect earlier and potentially clinically actionable stages, there are challenges obtaining adequate tissue volumes for research due to the safety concerns. Combined, autopsy and biopsy tissue have been key to understanding the entirety of DIPG pathogenesis and has substantially increased our understanding of this disease.

### Establishment of Cell Culture and Xenograft Models

Propagation of DIPG cells can be accomplished through *in vitro* expansion (indirect) or transplanting the tissue for animal xenograft (direct). The majority of tissue samples are first propagated by *in vitro* neurosphere cultures, once cells have been sufficiently expanded and the cell line is stable, then an indirect xenograft may be attempted. Immortalization of DIPG cells with hTERT (human telomerase ribonucleoprotein reverse transcriptase) has been used as an optional technique to establish DIPG models. The hTERT-modified cells are tumorigenic in athymic rodents and produce brainstem tumors that recapitulate the infiltrative brainstem gliomas (25). Although highly successful, the cell culture derived xenograft approach has some limitations. Notably, exposure of cells to tissue culture and exogenous growth factors can result in fundamental genetic and epigenetic changes to these tumor cells.

Considerable effort has been made to create direct models by injecting fresh DIPG cells directly into animals (18). While successful at times, the direct xenografts of DIPG cells are not without potential caveats: in one study, direct xenografts led to induction of murine tumors resembling DIPG (26). Furthermore, this method uses considerably more tissue and risks valuable tissue losses (18).

One primary consideration in creating xenograft models is to choose between heterotopic or orthotopic placement of the grafts. For heterotopic subcutaneous models, the cells are implanted between the dermis and underlying muscle typically on the flank of the mouse. Establishing subcutaneous xenografts are generally an easy procedure and often sufficient to test anti-cancer drugs. However, the native location of this tumor is in the brainstem and it is possible not being in this native anatomic milieu will have deleterious effects on maintaining the DIPG phenotype. In addition, drug regimens that are effective in subcutaneous xenograft models are frequently not efficacious in the brain because they do not pass the blood brain barrier (BBB) (27).

Orthotopic xenografts (tumor cells implanted into the equivalent organ of cancer origin) are preferred due to their clinical relevance. Advantages of orthotopic models include use of the relevant site for tumor-host interactions, the ability to study site-specific dependence of therapy, organ-specific expression of genes and replication of clinical relevance. However, establishing orthotopic xenografts of DIPG is technically more challenging, requiring longer recovery times and more sophisticated techniques to monitor tumor burden.

Whether success of DIPG engraftment varies among various immuno-compromised animal models is yet to be determined. Monje and colleagues stereotactically implanted patient-derived DIPG tumor cells into the pons of non-obese diabetic/SCID/γ-chain null–immuno-deficient mice to create the first patient-derived DIPG xenograft model (19). While most groups now use athymic nude mice (inhibited immune system with reduction of number of T cells only) for the engraftments as they are significantly less immuno-compromised while still enabling the engraftment, growth and eventual metastasis of tumor cells from the xenograft (25, 28).

### Genetically Engineered Models

Genetically engineered mouse models (GEMMs) recapitulate many of the common mutations found in human tumors and allow temporal and spatial assessment of tumor formation in immune competent animals (29). An early GEMM model of DIPG was generated using the replication-competent avian sarcoma-leucosis virus (RCAS) vector to enable Ink4a-ARF loss and platelet-derived growth factor B (PDGFB) overexpression within nestin-expressing cells in the pons of genetically engineered pups expressing tumor virus A (TVA) under the nestin promoter. This strategy successfully induced infiltrative gliomas in the mouse brainstem that were not exclusive to pons (30, 31). These GEMM models have been used for preclinical evaluation of targeted agents (32). A more recent DIPG GEMM model utilizes DIPG specific genetic alterations *PDGF-B, H3.3.K27M*, and *p53*. The DF1 (chicken fibroblast) cells modified with RCAS-PDGFB, RCAS-Cre and RCAS-H3.3.K27M delivered into the brain stem of mice pups engineered to express n*estin-TVA;p53*^*fl*/*fl*^, thus allowing conditional creation of DIPG-like tumors in the mouse brainstem (30). The tumors that developed in these animals demonstrate loss of H3K27me3 and express H3K27M mutant protein and therefore recapitulate the characteristic phenotype/genotype of human DIPG tumors.

Other approaches to develop H3K27M tumors in mice have been utilized including an approach with human embryonic stem cell derived neural progenitor cells (NPCs). Mimicking characteristic *PDGFR-A* modification through a constitutively active form D842V, shRNA knockdown of *p53* and inducing H3.3K27M mutation through lentiviral modification, Funato and coworkers created a stable and expandable system that resulted in brain tumor formation when injected into the pons of mice (33). Similarly, Mohammad et al. used modified mouse neural stem cells expressing the H3K27M mutation combined with *PDGF-B* and upon transplantation to SCID mice pons, these cells formed DIPG-like tumors as characterized by H3K27 hypomethylation in addition to Nestin, Olig2, and Atrx expression (34). An additional approach to cell line modification includes *in utero* electroporation. Newer studies using CRISPR methodology similarly find that H3K27M and p53 deletion replicates glioma formation. However, in contrast to previous study utilizing nestin-based expression, the authors find that such combination was unable to cause glioma formation (35, 36). While genetically engineered cell lines and mouse models are highly useful in preclinical evaluation of therapeutic agents and understanding the pathophysiology of the H3K27M mutation, these models lack the molecular heterogeneity of patient's tumors and may have epigenetic landscapes dissimilar from that of human DIPG tumors.

### Techniques for Assessment of DIPG Models

**Figure 1:**
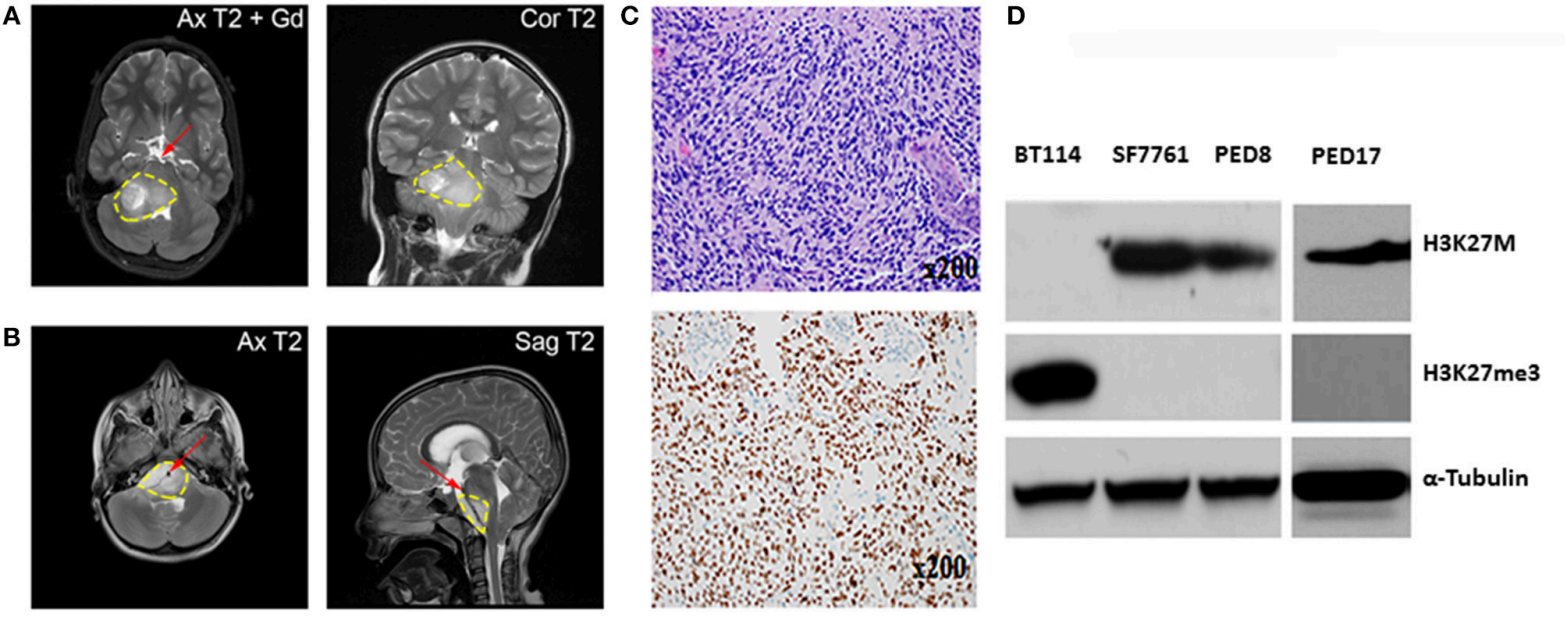
Characterization of Mayo patient-derived DIPGs. **(A**,**B)** MRI shows a large infiltrative variably enhancing T2 hyperintense brainstem mass (yellow contour line). **(A)** Axial and coronal T2 images of the patient from which PED17 was derived (Gd, gadolinium). Image shows pontine mass extending into midbrain and medulla with exophytic right cerebellar component. **(B)** Axial and sagittal MRI of a large infiltrative DIPG tumor extending from medulla to thalami with encasement of basilar artery (red arrow). **(C)** Above: Patient biopsy sample (Hematoxylin and Eosin) which produced PED17 cell line, showed a hypercellular glioma, composed of cells with astrocytic morphology, brisk mitotic activity, microvascular proliferation, and tumor necrosis. Below: Immunohistochemistry for H3K27M showed strong nuclear staining in more than 80% of tumor cells. **(D)** Western Blot showing expression of H3K27M, and H3K27 trimethylation (H3K27me3) in whole cell lysates of DIPG patient derived cell lines (SF7761, PED8, and PED17) and adult glioblastoma cells (BT114), α-Tubulin was used as loading control.

**Figure 2:**
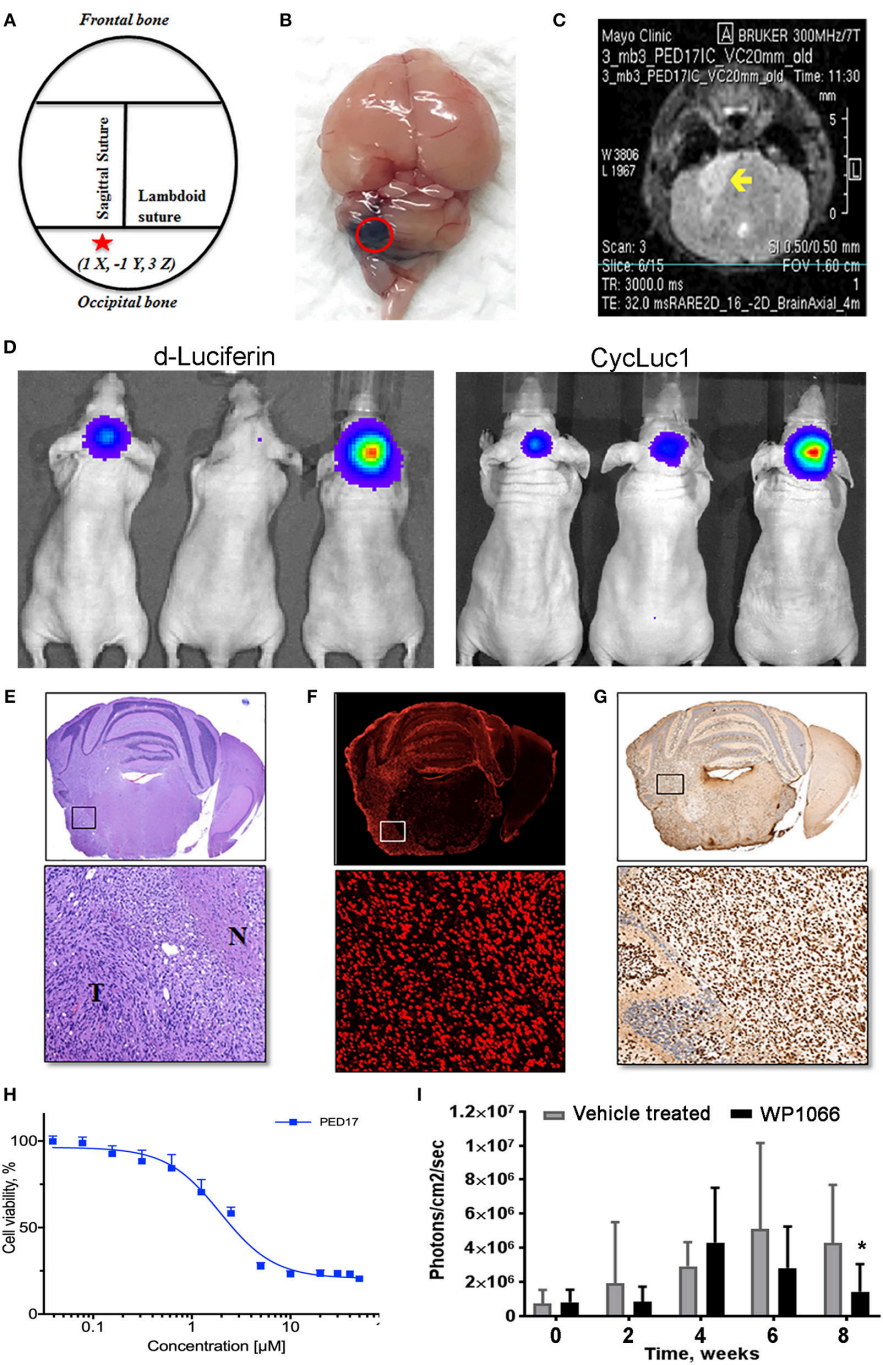
PED17-G-L tumor development and use for new therapeutic applications. **(A)** Standard coordinates were used to target the pons of mice: 1 mm inferior to the lambdoid suture and 1 mm lateral to the sagittal suture and 3 mm in depth. **(B)** Trypan blue (dye) injection into the pontine tegmentum of athymic mice (3μl) shows the area of distribution in the mouse brain (dorsal view) at the level of the pons. **(C)** Axial T2 MRI scan of PED17-G-L orthotopic xenograft. Tumor (yellow arrow) is seen as a hyperintense area in the right lateral pons. **(D)** PED17-G-L tumor progression assessed by BLI: d-Luciferin (50 mM) and CycLuc1 (5 mM). **(E)** Pathological analyses of PED17-G-L tumor: H&E staining of axial section of mice brain (same mouse as MRI; **C**). Insert (x20): the tumor mass (T) and the infiltration to the normal brain tissue (N). **(F)** Immunofluorescence: Human PED17-G-L cells preserved their origin after implantation to the mouse brain (positive for Lamin A+C staining, insert, x20). **(G)** H3K27M Immunohistochemistry: PED17-G-L cells exhibit nuclear H3K27M staining (insert, x20). **(H)**. Dose response curve of PED17 treated with various concentrations of WP1066 for 72 h. Cell viability was determined using Cell Titer Blue (Promega). Values are the means ± S.E.M (error bars) of duplicate experiments (*n* = 6). **(I)** BLI was used to track tumor size in orthotopic xenografts with PED17-G-L tumors treated with WP1066 (20 mg/kg) vs. control. The differences in PED17-G-L tumor sizes (based on BLI) between Vehicle- and WP1066-treated tumor-bearing animals (10 mice/group) after 6 weeks of treatment were statistically significant (^*^p = 0.028, *t*-test, difference between Vehicle and WP1066-treated tumors).

In patients, DIPG is usually diagnosed radiographically by an enlarged and swollen pons with encasement of the basilar artery (Figure 1), however, profound variability exists (4, 13). T1-weighted MRI sequence typical may exhibit hypointensity, whereas T2 (Figure 1) signal shows hyperintensity with variable contrast enhancement. However, MRI is not a fully reliable method to assess DIPG tumor formation and progression in mouse xenografts because of the small physical size, diffuse, and infiltrative nature of the tumor and variable contrast enhancement. Furthermore, the significant cost of MRI precludes its general use in research. Notable recent attempts have been made to develop other imaging modalities including positron emission tomography to assess animal xenografts (37, 38) and pediatric DIPG (39). Bioluminescence Imaging (BLI) is a commonly used non-invasive imaging technique to visualize tumor xenografts *in vivo*. However, to enable BLI, requires modification of tumor cells to express light emitting luciferase reporters that necessitates *in vitro* cell culture and thus not applicable to direct xenograft methods. DIPG xenografts using patient derived cell lines and short-term explants of patient-derived xenograft (PDX) lines have successfully used BLI to study *in vivo* tumor growth and drug efficacy (18). However, the commonly used BLI substrate, d-Luciferin has poor brain penetration, a critical variable to consider because of an intact BBB in DIPG tumors. The use of synthetic luciferase substrate, CycLuc1, which has better light emission property and unrestricted distribution across the BBB (40), can improve BLI for all brain tumors including DIPG. Our experience using CycLuc1 for BLI showed a significant signal enhancement and more persistent light emission than with d-Luciferin (Figure 2D).

DIPG xenografts and GEMM tumors are ultimately characterized by histopathological examination after termination. Similar to patient tumors (Figure 1C), diffuse spread of engrafted glioma cells can be seen on hematoxylin and eosin (H&E) slides (Figure 2E). As previously demonstrated, disseminated tumor cells are the hallmark of DIPG tumors (41). Immunohistochemistry (IHC) for H3K27M mutant proteins and loss of H3K27 trimethylation are now commonly used to assess mutation retention in DIPG xenograft tissues (Figures 1D, 2G).

## PED17, A Mayo Clinic Patient-Derived DIPG Models

Since January 2015, with Institutional Review Board approval, tissue biopsies from over 40 pediatric brain tumors patients, including 10 DIPG samples have been obtained at the Mayo Clinic. These tumor samples have been placed directly into cell culture in Multipurpose Handling Medium (MHM), or directly into the flank of athymic mice, or both if there were enough samples. Tissues were minced and passed through a Falcon cell strainer to prepare single cell suspension and attempts were made for direct engraftment in the flank of 6–7 weeks old female athymic nude mice (Envigo) with help from Glioblastoma PDX National Resource laboratory at Mayo Clinic. For detailed methods please refer to online Supplementary Material and Methods. All animal experiments were pre-approved by the Institutional Animal Care and Use Committee (IACUC) and conducted in accordance with IACUC guidelines. While none of the DIPG patient samples tumors could establish flank xenografts directly, we successfully created three patient derived cell culture lines with the H3K27M mutation from 3 patient samples (PED8, PED17, and PED36): these cell lines proliferated, formed neurospheres, and could be sub-cultured repeatedly. Confirming DIPG identity, the characteristic H3K27M mutation and loss of H3K27 trimethylation was observed in all our established cultured cell lines consistent with the parent tumors (Figure 1D).

Our cultured cell lines were able to establish indirect orthotopic xenografts in mice and their pathological characteristics, including infiltrative growth, were confirmed by histological examination. Here we summarize our experience with one of these lines, PED17 DIPG, as it has become one of our most reliable lines for *in vivo* and *in vitro* work. Cells were originally obtained from a surgical biopsy (Figure 1A) of an 8 year-old female's DIPG tumor. The patient presented with a 6-week history of headaches and double vision. Subsequent histopathological examination of the biopsy tissue showed a hypercellular infiltrating cells with an astrocytic morphology, harboring brisk mitotic activity, microvascular proliferation, and a necrotic core (Figure 1C), supporting the diagnosis of glioblastoma (WHO grade IV) per the 2007 WHO Classification of CNS Tumors. Genomic testing revealed five alterations: *H3F3A* K27M, *ATRX* loss, *TP53* (R282W), *PTEN* loss and *CDK4* amplification but no mutations in *PDGFRA, IDH1*, or *EGFR*. Since 2016, WHO classifies this tumor type as Diffuse Midline Glioma, H3K27M mutant.

After three passages in culture we confirmed the cells still harbored the H3K27M mutation and loss of H3K27 trimethylation (Figure 1D); then, PED17 tumor cells were labeled with a GFP-LUC expression construct using lentiviral transduction (see Material and Methods). The luciferase tagged cells, PED17-GFP-LUC (PED17-G-L) were then stereotactically injected in the pons of 6–7 weeks old athymic female mice using standard coordinates for murine pons injections (Figures 2A,B) (42). Sixteen weeks after implantation, MRI imaging was performed using a 300 MHz (7 Tesla) large vertical bore NMR spectrometer that can accommodate mice (see Materials and Methods). As expected, axial T2 MRI demonstrated hyper-intense areas in the pons in the general region of the injections (Figure 2C), which was hypointense on T1 and lacked gadolinium contrast enhancement. However, due to the small size and resolution of the instrument, the degree of tumor infiltration through the pons is not adequately imaged with this modality as we ultimately observe significant tumor infiltration through the entire pons and brainstem on pathologic examination (Figures 2E–G). PED17-G-L tumor engraftment and progression was assessed by BLI using the brain penetrant CycLuc1 instead of d-Luciferin as it produced longer and more intense BLI signal (Figure 2D).

To confirm whether xenograft tissue retained the primary tumor characteristics, the animals were assessed by histopathological examination by a neuropathologist (Dr. Raghunathan, Mayo Clinic). Animals were euthanized, whole brains were surgically removed and fixed in 4% paraformaldehyde, embedded in paraffin and sectioned in the axial plane. Under the microscope, H&E revealed that PED17-G-L xenografts had developed highly infiltrative tumors throughout the brainstem similar to patient tumors. Morphologically, highly cellular and poorly differentiated tumors composed of monotonous large and rounded cells with central nuclei and prominent nucleoli (Figure 2E). IHC for H3K27M showed strong positivity throughout the entire pons (Figure 2G). Since other researchers (Caretti et al.) have previously reported that human DIPG xeno-transplatation may lead to induction of murine tumors resembling DIPG tumors (26), we carefully examined the origin of these tumor cells. Immunofluorescence staining with human-specific Lamin A+C confirmed the xenografted cells were of human origin (Figure 2F). Standard resolution MRI was not able to capture the degree of infiltration in these orthotopic xenografts (Figure 2C), the diffuse pathology was seen on H&E and IHC (Figures 2E–G). Likely the diffuse nature of DIPG xenografts and small anatomic size, make standard MRI (7T) less useful for monitoring tumor growth and progression in mice as it may be difficult to discern infiltrative tumor cells from normal brain.

### Therapeutic Developments Using Patient Derived H3K27M Models

Using our patient derived H3K27M cell lines we have performed rigorous high-throughput drug and shRNA knockdown screens to identify potential molecular targets. By using multiple H3K27M and wild type cell lines, initial targets were identified from compounds that selectively reduced cell viability in H3K27M vs. wild type cells. Compounds of interest were then assessed based on their efficacy and ability to cross the BBB. Key compounds then underwent *in vivo* testing in our PDX murine models. As an example, the tyrosine kinase inhibitor WP1066 was tested *in vitro* (Figure 2H) using CellTiter-Blue viability assay.

Orthotopic PED17-G-L treatment with oral WP1066 was initiated 6 weeks post tumor implantation into the pons of athymic nude mice in two groups (10 mice/group): vehicle and WP1066 treatment (20 mg/kg). The inhibitor was provided 3 times a week every other day for 6 weeks by oral gavages. Animals were monitored daily and euthanized at indications of progressive neurologic decline including observed postural and balance deficits, or if found in a moribund condition including weight loss and seizure activity. WP1066 significantly reduced H3K27M PED17-G-L tumor growth in orthotopic xenografts compared to control at 8 weeks (Figure 2I). These results demonstrate the utility of orthotopic xenograft models and provide rationale for clinical evaluation of WP1066 in patients with DIPG.

## Conclusion and Future Directions

Recent surgical advancements and willingness to obtain DIPG tumor tissue has facilitated a new era of research for this deadly disease. With refinement of both, patient derived xenograft models and complementary genetically engineered animals, newer models that more accurately recapitulate the genetic and phenotypic hallmarks of DIPG tumors have been created. Importantly, such models have advanced our understanding of this disease. With assessment of new key markers including H3K27M, loss of H3K27 trimethylation, modern DIPG research is primed for clinical translation. Only through the continued advancement of these models may we begin to find effective therapies for this devastating disease.

## Author Contributions

JW, TK, and DD: conception and design; JW, TK, AW, LZ, TEP and AR: analysis and interpretation of data; JW, TK, SG, DB, and AR: drafting the article; DD: reviewed submitted version of manuscript, approved the final version of the manuscript on behalf of all authors and study supervision. All authors acquisition of data and critically revising the article.

### Conflict of Interest Statement

The authors declare that the research was conducted in the absence of any commercial or financial relationships that could be construed as a potential conflict of interest.
