# Supplementary material for: Current Murine Models and New Developments in H3K27M Diffuse Midline Gliomas
Source: Front Oncol. 2019 Feb 27;9:92. doi: 10.3389/fonc.2019.00092 (PMC6400847; doi:10.3389/fonc.2019.00092)
Supplement: Supplementary file 1 [file Data_Sheet_1.docx]

Supplementary Material

**MATERIALS AND METHODS**

**Isolation and Culture of Pediatric DIPG Cells**

The Mayo Clinic and Foundation Committee on Ethics of Biomedicine Research and Institutional Review Board approved the study protocol and acquisition of tissue specimens. Dr. D. J. Daniels Research Laboratory, Departments of Pediatric Neurosurgery, Mayo Clinic, Rochester, MN obtained surgical tissue specimens. The tissue was minced into small pieces and passed through a Falcon cell strainer. After washes in PBS, cells were cultured as a neurospheres in MHM medium (DMEM/F12 1:1 (HyClone#SH30023.01) supplemented with 1.2% NaHCO3 (Gibco#25080094), 1% Glucose (Sigma#G8769), 1% Glutamax (Gibco#35050-061), 1% Pen/Strep (Gibco#15140122), 1% N2 Supplement (Gibco#17502048), 0.4% HEPES (MP#1688449), 20 ng/ml h-b-FGF (Peprotech#AF-100-18B), 20 ng/ml h-EGF (Peprotech#AF-100-15), 4 mg/ml Heparin Sodium Salt (Sigma#H3149), and 10 ng/ml h-PDGF-AA&BB (Shenandoah Biotech#100-16&18).

**Lentiviral transduction**

After three passages, the cells were transduced with a lentiviral vector pRRLSIN-18.PPT.LUC-GFP.pre (kind gift from Dr. Monje’s lab (Stanford, CA)) that was packaged into lentiviral particles by co-transfecting 293T cells with the mixture of pxPAX2 (Addgene) and pMD2.G (Addgene) plasmids (Lin, G.L., and Monje, M. (2017). A Protocol for Rapid Post-mortem Cell Culture of Diffuse Intrinsic Pontine Glioma (DIPG). (121)**,** e55360. doi: doi:10.3791/55360.). Lentiviral particles collected and were concentrated by polyethylene glycol precipitation solution (BioCat) following packaged instructions and resuspended in PBS and used for transduction of PED17 cells (the efficiency was evaluated by fluorescence microscopy).

**Development of cell-derived orthotopic xenograft**

After about 30 days in culture, PED17-GFP-LUC (PED17-G-L) DIPG cells were stereotactically injected into the Nude mice. Female Hsd:athymic Nude Foxn1nu mice (age 6–7 weeks; Envigo) were kept under specific pathogen-free conditions in air-filtered cages and received food and water *ad libitum.* The mice were handled in accordance with IACUC guidelines and the Mayo Clinic Institutional Committee for Animal Research approved all the experiments. The PED17-GFP-LUC xenograft model was established by intracranial transplantation of 3 × 10^5^ cells/3µl of PBS, after <5 passages *in vitro.* The cells for injection were resuspended in sterile PBS at 100,000 cells/μL in a 1.5-mL sterile microcentrifuge tube. For stereotactic surgery mice were sedated using 10% of Ketamine and 5% of Xylazine mixture in sterile PBS. For a 20g mouse, use approximately 200 μL of the ketamine/xylazine mixture injected intraperitoneally with a 0.5-cc syringe for a dose of 100mg/kg ketamine and 10mg/kg xylazine. A total of 3× 10^5^ of PED17-G-F cells were injected stereotactically in a volume of 3 µL. The skull of the mouse was exposed and a small burr hole (0.5 mm) was made using a #7 or #8 bit in a high-speed Dremel drill at the follow stereotactic coordinates: 1 mm inferior to the lambdoid suture and 1 mm lateral to the mid-sagittal plane and 3 mm in depth (Fig. 2A&B). PED17-G-L cells were injected at a speed of 1 µL per minute into the pons with a 26-gauge (2 mm, point style AS) Hamilton syringe (Bonaduz, GR, Switzerland). After closing the scalp, mice were placed on a warming pad and returned to their cages after full recovery. Mice with intracranial tumors were observed daily since neurologic decline can occur rapidly, at which point mice were euthanized. Typical signs of neurologic decline included a hunched posture with an arched back, circling, walking on their tip-toes, balance issues, eyes that are not opened, weight loss, hyperactivity and seizures.

***In vivo* Bioluminescence Imaging (BLI)**

PED17-G-L tumor engraftment and progression was also assessed by BLI. For BLI experiments, animals were injected intraperitoneally with 100 µl solution of either 50 mM d-Luciferin (Gold Biotechnology) or 5 mM CycLuc1 (Millipore), bioluminescence acquired using the IVIS-2000 Imaging System (Xenogen Corporation, Berkeley, CA) and analyzed by LivingImage 4.3 software. Two minutes after d-Luciferin administration, all 3 animals presented bioluminescent signal (data not shown), however, BLI signal in one of 3 mice was significantly diminished overtime and not detectable at 30 min (Fig. 2D). In contrast, CycLuc1 mediated BLI provided a stable signal over 30 min for all animals. These results suggest that in PED17-G-L cells CycLuc1 is a good BLI substrate and that BLI can reliably detect orthotopic xenografts of DIPG. Signal intensities were quantified within regions of interest, as defined by the LivingImage software. Bioluminescence measurements for each animal at each time point was normalized against corresponding readings obtained at the beginning of therapy. The peak signals were compared using unpaired 2-tailed *t*-test. Comparisons where p<0.05 were considered statistically significant.

***In vivo* Magnetic Resonance Imaging (MRI)**

Mice were placed under anesthesia using 1.5-2.5% isoflurane to immobilize the animal before and during the MRI scan. MRI was performed in a Bruker Avance 300 MHz (7 Tesla) vertical bore NMR spectrometer equipped with “mini-imaging” accessories. The 37°C core temperature of the animals were maintained by a stream of air conditioned by thermocouple based system at 32°C. Inhalational isoflurane anesthesia (1.5%-2.5% in oxygen/air) were delivered via a nose cone during the imaging procedure. Animal’s respiration was continuously monitored during the scan and was kept at 20-60 rpm by adjusting percentage of the isoflurane. The duration of anesthesia was less than 2 h at a time.

**Immunohistochemical staining (IHC)**

We assessed the maintenance of histone H3K27M mutation in PED17-GFP-FLUC xenografts by Immunohistochemistry (IHC). Animals were euthanized by cervical dislocation and the brains were fixed in 4% paraformaldehyde for 72h at RT, sectioned in the coronal plane at 40 μm using a sliding microtome (Microm HM450; Thermo Scientific). Sodium Citrate Buffer (10mM Sodium Citrate, 0.05% Tween 20, pH 6.0) was used as an antigen retrieval reagent prior to the blocking step. For immunohistochemistry, coronal sections were incubated in blocking solution (10% Normal Goat Serum in PBS) at room temperature for 30 minutes. Rabbit mAb Anti-Histone H3 (mutated K27M) (Abcam#190631) and Rabbit anti-human Lamin A+C (Abcam#108595) antibodies were diluted 1:200 in antibody solution (1% normal goat serum in 0.3% Triton X-100 in PBS) and incubated 1h at RT. Sections were then rinsed three times (10 min) in antibody solution and incubated with Goat anti-Rabbit IgG [Cy5] Secondary Antibody (1:200) (Novus#NB120-6564) for 1h at RT. Sections were then rinsed thrice in antibody solution and mounted with ProLong DAPI Mounting medium (Life Technologies).

***In vivo* administration of WP1066 (STAT3 Inhibitor)**

WP1066 treatment (20mg/kg) of PED17-G-L xenografts was initiated after 6 weeks of the tumor intracranial implantation at the cohorts of the 10 mice in both groups, Vehicle- and WP1066-treated. STAT3 Inhibitor treatment was provided 3 times a week every other day for 6 weeks as an oral gavage (500 μg WP1066/100 μL of mixture of 20 parts DMSO: 80 parts PEG300/25g mouse). Animals were monitored daily and euthanized at indication of progressive neurologic deficit or if found in a moribund condition.

**Statistical Analysis**

All statistical analysis was performed using a two-tailed Student’s t-test with a threshold alpha p<0.05.
